# The Stigma of Hearing Loss: A Scoping Review of the Literature Across Age and Gender

**DOI:** 10.1002/ohn.1246

**Published:** 2025-04-09

**Authors:** Caroline Liu, Maria M. Mavrommatis, Aparna Govindan, Maura K. Cosetti

**Affiliations:** ^1^ Department of Otolaryngology–Head and Neck Surgery University of Nevada, Las Vegas Las Vegas Nevada USA; ^2^ Department of Otolaryngology–Head and Neck Surgery Icahn School of Medicine at Mount Sinai New York New York USA; ^3^ Department of Otolaryngology–Head and Neck Surgery University of Miami/Jackson Health System Miami Florida USA

**Keywords:** hearing aids, Hearing Health Collaborative, hearing loss, scoping review, stigma, utilization

## Abstract

**Objective:**

Stigma is a human construct that guides community standards and opinions, often characterized by negative beliefs about a particular circumstance, quality, or person. This study reviews the literature for stigma related to hearing loss and hearing device use.

**Data Sources:**

PubMed, Scopus, and Embase.

**Review Methods:**

Databases were searched from inception to April 28, 2024. Two independent researchers screened articles and performed full‐text reviews. Grounded theory was used to identify and analyze positive and negative themes across disparate qualitative data.

**Results:**

After screening 1096 abstracts, 45 full‐texts and 4 conference abstracts were included including 17 studies in pediatric populations, 19 studies in adults of working age, and 14 studies in older adult populations. In pediatric populations, stigma is primarily tied to bullying and poor classmate perceptions, with school‐based supports offering mixed results in minimizing perceived stigma. Among working and older age adults, common positive themes included improved quality of life and self‐empowerment among hearing aid (HA) users. All working age studies refer to the role of HAs in creating a visible disability. The pervasive theme among older adults was being perceived as old or senile. Although studies were largely equal in gender representation, differential gender effects of stigma and HA decisions were identified.

**Conclusion:**

Hearing loss stigma appears to be pervasive across age and gender with distinctions that have implications for intervention development. Future studies are needed to parse further nuances related to the stigma of hearing loss.

Hearing loss affects approximately 50 million individuals in the United States.^1^ As the US population ages, hearing loss is expected to increase to 26% (74 million) by 2060^2^ with estimates as high as 67.4% among adults older than 70 years, and more than >80% among adults older than 80 years.^3^ Hearing loss can affect all ages with prevalence estimates as high as 20% among US adolescents aged 12 to 19.^4^, ^5^ In school‐aged children, hearing loss can significantly impact speech and language development and subsequent educational and social development.^6^ Given the consequences, hearing screening in newborns and children is mandated across the United States.^7^

Despite the ubiquity of hearing loss, less than one‐third of American adults with hearing loss use hearing aids (HAs)^8^ often attributed to barriers such as cost and comfort.^9^ Multiple countries have attempted to minimize socioeconomic barriers through government and insurance coverage without success in increasing HA adoption rates. In Germany, HAs are fully covered for incomes less than €60,000 (US $72,856).^10^ However, the HA usage is estimated to be only 37%.^10^ In Iceland, HA usage rates are less than 25% despite full coverage of HA costs.^11^, ^12^

Low usage rates despite cost reduction efforts suggest that psychosocial factors, such as stigma may influence HA decisions. On August 16, 2022, the Food and Drug Administration (FDA) established a regulatory category for over‐the‐counter (OTC) HAs to improve HA access in the United States.^13^ However, just a few months prior, Bose™ withdrew from the HA market citing a poor consumer market for HAs due to a “three‐body force…of stigma, denial, and apathy.”^6^ Characteristics such as perceived benefit, locus of control, and personality traits may contribute to a person's decision to pursue treatment for hearing loss and should be accounted for in health policy development.^9^

Hearing loss is not the only medical condition adversely affected by stigma. The field of endocrinology termed the phrase “adiposity‐based chronic disease” to capture the pathophysiologic basis of the condition and avoid stigmata from the term “obesity.”^14^ At present, no such parallel exists in hearing health care. As such, we aimed to characterize the current literature on hearing loss stigma.

## Methods

We conducted a scoping systematic literature review on stigma surrounding hearing loss and uptake of hearing loss technology (HAs and cochlear implants [CIs]). We searched PubMed, Scopus, and Embase from database inception to April 28, 2024, and cross‐referenced study bibliographies. The full search criteria can be seen in Supplemental Table S1, available online.

C.L. and M.M.M. screened abstracts, full texts, and abstracted the data. We included studies on stigma, social perceptions, or perceived discrimination in persons with hearing loss HA or CI users. All primary study designs were included without restrictions on sample size. We included all age groups. Prior reviews have excluded pediatric studies out of concern for differential relationships between stigma and hearing technology in populations who exclusively use manual communication.^15^, ^16^, ^17^ Given the general paucity of pediatric hearing loss stigma literature, we included pediatric studies so long as the study populations were not exclusive to manual communication. We excluded prior reviews and studies unavailable in English text or after a comprehensive library request. This review was performed in accordance with the Preferred Reporting Items for Systematic Review and Meta‐Analyses Extension for Scoping Reviews to assess the following question: In all available original study designs among patients with hearing loss, does stigma affect the decision to pursue hearing loss treatment?^18^

Data were then evaluated using grounded theory, a systematic methodology that has been applied to qualitative research to identify, organize, and analyze themes around a larger research question.^19^ A thematic analysis was performed based on grounded theory by two reviewers (C.L. and M.M.M.) and then extracted directly from each study. We analyzed positive and negative “themes” within each study.

## Results

**Figure 1:**
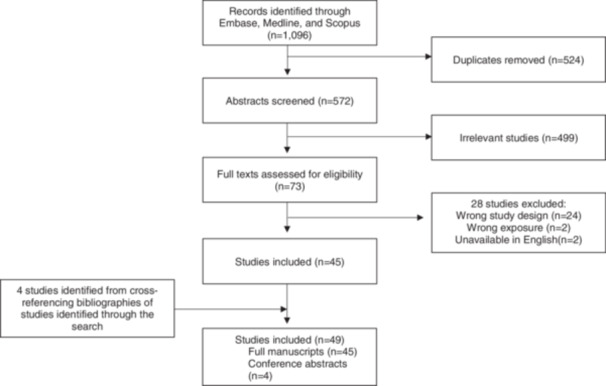
Flowchart of study inclusion.

We identified a total of 1096 abstracts (Figure 1) with 572 articles available for screening after removal of duplicates (n = 524). We screened 73 full texts and included 45 studies in the review (24 studies were excluded due to incorrect study population or if the article was a research meeting abstract later published as a full manuscript [subsequent full text included], 2 for wrong exposure, and 2 due to unavailability of English text). Four studies were identified by cross‐referencing bibliographies. Of the total 49 studies included, 45 (92%) were full manuscripts and 4 (8%) were conference abstracts.

### Stigma and Hearing Loss in Pediatric and Adolescent Populations

Sixteen pediatric studies were included (Supplemental Table S2, available online).^20^, ^21^, ^22^, ^23^, ^24^, ^25^, ^26^, ^27^, ^28^, ^29^, ^30^, ^31^, ^32^, ^33^, ^34^, ^35^, ^36^ The role of stigma and hearing loss in pediatric populations is two‐fold: (1) the perception from the child and (2) the concern for stigma by the child's caregivers. Among the 16 studies identified, 4 studies examined both child and caregivers’ perceptions, 4 studies were conducted among caregivers only, and 8 were conducted exclusively among the children and adolescents. Most studies consisted of thematic analysis of interviews (n = 8). Four standardized metrics were employed: the Youth Quality of Life Instrument–Deaf and Hard of Hearing (YQoL‐DHH),^27^, ^29^ DISABKIDS,^20^, ^33^ KIDSCREEN,^33^ and the Health Behavior in School Aged Children (HBSC)^23^ questionnaires.

Stigma in pediatric populations appears tied to concern for bullying and peer perceptions in school‐based settings. In all eight interview‐based studies, pediatric participants commonly reported socially induced perceptions of being an “abnormality” owing to the visibility of HAs.^21^, ^23^, ^25^, ^26^, ^30^, ^31^, ^33^, ^36^ Kent found that most surveyed students with hearing loss (29/52 [55.8%]) did not self‐identify as having a hearing disability. Those who self‐identified as having a hearing disability were statistically more likely to report loneliness and bullying.^23^ The authors hypothesize that the reluctance to self‐identify could be due to perceived negative stigma or differences in opinion of hearing loss as a disability.^23^

Using grounded theory, the following positive “themes” of HA use were identified across pediatric studies: ease of use, positive feelings toward school and learning, and self‐empowerment. Negative themes of HA were far more varied, including visibility and cosmetics (12/17 papers), exposure to classmate's curiosity (12/17 papers), bullying (9/17 papers), fear of rejection (5/17 papers), limitation in participation of daily activities (3/17 papers), lack of environmental support (3/17 papers), lack of self‐identification of a disability (2/17 papers), and loneliness (1/17 papers) (Supplemental Table S2, available online).

Age‐matched children and adolescents without hearing loss tended to have negative perceptions of their peers with hearing loss.^21^, ^23^, ^24^, ^26^, ^34^ Qian et al compared perceptions of age‐matched children on peers who wore HAs and glasses.^34^ Unlike glasses, which were perceived as a mark of intelligence, age‐matched peers without hearing loss perceived HA users unfavorably with regard to athleticism, confidence, health, intelligence, leadership, popularity, and success.^34^ Negative perceptions were stronger for those with brightly colored HA wearers compared to those with neutral colored HAs. Furthermore, brightly colored HAs in conjunction with glasses negated the perceived intelligence of glasses.^34^ Strange et al^26^ and Ryan et al^24^ reported similar findings where the more visible the HA, the more negatively age‐matched peers without hearing loss viewed the HA user.

Interventions to support children with hearing loss and HAs in classroom settings have mixed effects. Strange et al found that a brief intervention explaining the benefits of HAs provided some desensitization to the biases against HA users, reducing internalized stigma.^26^ However, classroom‐based supports such as preferential classroom seating may adversely impact peer perceptions.^30^ In a sample of 16 teenagers with unilateral hearing loss, 12 (75%) reported concerns with sitting at the front of the class and hesitancy to request a new seating assignment.^30^ A simulated hearing loss session with subsequent HA fittings among normal hearing college students led to favorable perceptions of HAs.^21^ However, 25% would still opt out of HAs citing cosmetics and perceived stigma.^21^

Studies on caregiver perceptions of stigma related to pediatric hearing loss were limited in sample size and demonstrated mixed findings.^22^, ^28^, ^30^ Most parents reported minimal barriers in their child(ren)'s day‐to‐day HA use. In a cohort of 50 parents, a few parents cited stigma behind their decision to cease HA usage (1/50) or decline a device trial (3/50).^30^ In a cohort of 39 parents, only one parent declined HAs due to stigma.^22^ Although parents expressed fears surrounding the potential social stigma of HAs, few reported significant teasing due to HAs.^28^ Parents were challenged with weighing their concern for stigma against the consequences of uncorrected hearing difficulties.^22^

Two CI studies were included and were conducted in pediatric cohorts.^27^, ^32^ Among children with severe to profound sensorineural hearing loss, those with CIs reported less stigma and better quality of life than those with HAs.^27^ Chang found that normal hearing caregivers of children with severe sensorineural hearing loss felt stigma for pursuing CIs.^32^

### Stigma and Hearing Loss in Working Age Adults

We identified 19 studies on stigma and hearing loss conducted among working‐aged adults (Supplemental Table S3, available online).^31^, ^37^, ^38^, ^39^, ^40^, ^41^, ^42^, ^43^, ^44^, ^45^, ^46^, ^47^, ^48^, ^49^, ^50^, ^51^, ^52^, ^53^, ^54^ Across all studies, participants refer to the visibility of HAs or the implications of a visible disability.^38^, ^43^, ^46^, ^47^, ^53^ Data were obtained primarily through structured interviews (n = 17 studies). Three studies gathered quantitative data that were unamenable to pooling.^49^, ^51^, ^54^

Grounded theory identified the following positive themes of HA use: improvement in quality of life, positive impact on interpersonal relationships, self‐empowerment, self‐acceptance, reduced mental workload, locus of control, and an opportunity to educate others on hearing loss. Negative themes of HA were more varied, including peer perception of intelligence or adequacy (15/19 papers), visibility and cosmetics (11/19 papers), ageism (10/19 papers), isolation (2/19 papers), fear of rejection (1/19 papers), bullying (1/19 papers), lack of environmental support (1/19 papers), low self‐esteem (1/19 papers), wage gap compared to hearing colleagues (1/19 papers), and lack of familial acceptance (1/19 papers).

The working age group is a heterogeneous population with regard to onset, duration, and severity of hearing loss with expected differences in perceived stigma. Structured interviews revealed common themes closely related to ageism as participants primarily cited fear of appearing older with HAs. Preminger and Laplante‐Lévesque found that younger participants expressed hesitancy toward HAs and requested aids that were targeted toward a younger demographic.^47^

Stigma may impact wage earnings among individuals with hearing loss. Benito et al found that the majority of working age participants were unfamiliar with the Americans with Disability Act, Public Law 101‐336, which prohibits employment discrimination against individuals with disabilities.^48^ Their data documented a wage gap in adults with hearing loss—of which 40% was attributed to differences in educational attainment, experience, race/ethnicity, and marital status, suggesting that the remaining 60% reflect differences in unobservable characteristics such as communication skills, labor market discrimination, and stigma.^48^

### Stigma and Hearing Loss in Older Adults

We identified 14 studies^55^, ^56^, ^57^, ^58^, ^59^, ^60^, ^61^, ^62^, ^63^, ^64^, ^65^, ^66^, ^67^, ^68^ (Supplemental Table S4, available online) in older adults, 4 with quantitative results.^55^, ^56^, ^59^, ^64^ By far, the most commonly reported theme‐based analysis of interview transcript responses was a concern with being perceived as “old or senile.”^57^, ^58^, ^60^, ^61^, ^62^, ^63^, ^65^, ^66^, ^67^, ^68^ Positive themes of HA use included improvement in quality of life, self‐empowerment, community, and ease of access. Negative themes included visibility and cosmetics (8/14 papers), peer perception of intelligence or adequacy (7/14 papers), ageism (6/13 papers), fear of rejection (6/13 papers), self‐perception of weakness (5/14 papers), lack of self‐identification of disability (4/14 papers), and lack of environmental support (1/13 papers).

One of the largest studies published to date regarding stigma and HA usage included 4226 National Family Opinion panel members, representative of the US population with respect to market size, income, and age and size of household.^43^ HA adoption rates were substantially higher among older adults even after controlling for degree of hearing loss and income.^43^ Respondents older than 75 years had a ~four times greater chance of owning HAs than individuals ages 21 to 44 and twice that of 55‐ to 64‐year‐olds. Perceived stigma in this population was associated with entering a HA evaluation program (odds ratio [OR] [95% CI] 1.07 [1.00‐1.14]).^64^ Older adults with higher perceived stigma also had higher odds of reporting difficulty with inserting, using, or manipulating HAs (OR 3.48, *P* = .02).^56^

### Gender Differences in Stigma and Hearing Loss

Pediatric studies identified in this review equally represented children and adolescents of both genders. Ryan et al found that children tended to have negative perceptions against peers with hearing loss, regardless of gender.^24^ The majority of caregiver study participants were female, likely reflecting convenience sampling of caregivers present at children's health care visits.^24^, ^27^, ^28^

Among working age and older adult populations, we identified four studies conducted in female cohorts^39^, ^40^, ^41^, ^58^ with one focused exclusively on women older than 60 years.^58^ Consistent with co‐ed studies, Erler and Garstecki^41^ and Garstecki and Erler^40^ found that hearing loss stigma increased with age with the prevailing concern of being perceived as elderly. In two separate cohorts of women with normal hearing, younger women perceived greater stigma than older women.^40^, ^41^ Erler et al further identified reduced stigma *with* HA use, possibly suggesting that hearing loss management can have the opposite effect and reduce perceived stigma against hearing loss.^41^ Similarly, Hallberg and Jansson identified that perceived stigma against persons with hearing loss was associated with a higher odds of entering a HA evaluation period among female participants only.^39^ No significant relationship was identified among male participants.

### International Studies

In total, 29/49 (59%) studies were conducted in countries outside of the United States (Supplemental Table S5, available online) and were evenly distributed by age group: pediatric (n = 9), working age (n = 10), and older adults (n = 10). Most international studies were conducted in English‐speaking countries or took place in school settings where English was the primary language.^23^, ^24^, ^26^ Of note, Jaradeh et al conducted their US study among non‐English‐speaking caregivers.^36^ They found that children with non‐English‐speaking caregivers were more likely to experience delayed hearing loss diagnosis and treatment compared to their counterparts with English‐speaking parents.^36^ Both international and US studies bring into question the role of cultural and language differences in hearing loss stigma and the study designs used to study them.

## Discussion

This scoping review demonstrated that the bulk of literature on stigma in hearing loss and HA/CI use has been conducted through semistructured, qualitative interviews. Few studies used standardized questionnaires to report quantitative data with substantial variability across metrics limiting the summative ability across studies. Despite variation in stigma measurement, findings are largely consistent with pervasive findings of perceived and felt stigma among persons with hearing loss, with or without hearing treatment. This scoping review included all age groups to assess differential hearing loss stigma by age, with the inclusion of caregiver populations in the setting of pediatric hearing loss. Prior reviews have focused exclusively on older adults, with a focus on how hearing loss stigmata promotes social withdrawal and negative self‐perception.^16^, ^17^

The different facets of stigma by age group may have implications for age group‐specific interventions. In pediatric cohorts, the perceived and felt stigma of pediatric hearing loss is dual fold. As such, interventions regarding HA or CI decisions should target both the patient and their caregiver(s). Among older adults, hearing loss is detrimental to healthy aging with growing evidence linking hearing loss with cognitive decline.^69^ Lin et al found that HA use may reduce cognitive change among older adults at increased risk for cognitive decline.^70^ Early diagnosis and counseling of hearing loss rehabilitation should be approached as another modifiable risk factor for healthy aging.

Gender‐based differences in stigma have not been fully elucidated. Since 1993, the National Institutes of Health has mandated equal gender representation in clinical research. A prior search of ClinicalTrails.gov identified 564 hearing loss trials with an even gender distribution—ClinicalTrials.gov^71^ However, sociological topics such as stigma may have differential effects by gender as suggested by studies in the present review. A myriad of issues spanning the natural history of hearing loss to cosmesis may underlie differences between attitudes and behaviors of males and females related to HA adoption.

More than 20% of the included studies in this review were conducted over two decades ago. Since then, advances in HA technology have led to improved cosmesis and function. Ear‐mounted technology such as Bluetooth has made HAs less recognizable as a signpost of disability.^72^ In this review, cosmesis and visibility were cited as themes across all age groups. Among studies that investigate perceptions of HA esthetics, patients favor smaller and less visible HAs—a barrier that evolving technology may improve.

The visibility of HAs was negatively perceived across all age groups, in contrast to the positive stereotypes of glasses. The differential stigma between glasses and HAs can be attributed to historical associations of hearing loss as an impairment of reasoning and intelligence. Before modern technology, people with hearing loss could not participate in social interactions and became marginalized by society.^72^ These frameworks persist despite the rapid evolution of hearing technology and nonauditory communication.

The FDA's establishment of a regulatory category for OTC HAs aimed to address two barriers to hearing health care: cost and access to technology.^13^ However, the pervasive and unaddressed impact of stigma may continue to play an important role in the undertreatment of hearing loss. Further facets of stigma that warrant investigation include media and pop culture portrayals of persons with hearing loss and/or HAs, and health care settings. Future investigations into these topics are warranted and may have widespread implications for the adoption of HA technology across a variety of populations.

Our study has a number of limitations. Despite our attempts to be as broad as possible, our literature search may not have captured all relevant articles. To mitigate this, we cross‐referenced prior reviews and included studies. Our search was limited to Medline, Embase, and Scopus and may have benefited from adding sociological and psychological research databases. Subgroup analyses were limited by the number and sample sizes of included studies. Further subgroups such as caregiver populations, international cohorts, ethnic minority versus majority, English speaking versus non‐English speaking, socioeconomic status, and different hearing interventions (HAs vs CIs) warrant further investigation. Finally, most studies were qualitative in nature and not amenable to pooling.

## Conclusion

The stigma of hearing loss appears pervasive across age and gender. As the prevalence of hearing loss is expected to grow, improving our understanding of stigma will be critical in overcoming barriers to hearing health care across the lifespan.

## Author Contributions

**Caroline Liu**, conception of study design, acquisition of data, analysis and synthesis of data, and drafting of manuscript; **Maria M. Mavrommatis**, conception of study design, acquisition of data, analysis and synthesis of data, and drafting of manuscript; **Aparna Govindan**, conception of study design, acquisition of data, analysis and synthesis of data, and drafting of manuscript; **Maura K. Cosetti**, conception of study design, acquisition of data, analysis and synthesis of data, and drafting of manuscript.

## Disclosures

### Competing interests

All authors report no conflicts of interest concerning the materials, methods, or findings specified in this paper.

### Funding source

None.

## Supporting information

Supporting Information
