# Supplementary material for: The Stigma of Hearing Loss: A Scoping Review of the Literature Across Age and Gender
Source: Otolaryngol Head Neck Surg. 2025 Apr 9;172(6):1874–81. doi: 10.1002/ohn.1246 (PMC12120038; doi:10.1002/ohn.1246)
Supplement: Supplementary file 1 — Supporting Information [file OHN-172-1874-s001.docx]

# Supplemental Appendix

## **Supplemental Table 1. Search Criteria**

| **Database** | **Search Criteria** |
| --- | --- |
| PubMed | ("Hearing Loss"[Mesh] OR "Hearing Aids"[Mesh] OR "Persons With Hearing Impairments"[Mesh] OR (hearing impairment*) OR “hearing loss” OR “hearing aid” OR “hearing aids” OR “hard of hearing” OR Presbycusis)  AND  ("Social Stigma"[Mesh] OR stigma* OR "Social Perception"[Mesh] OR “social perception” OR “social perceptions” OR “Perceived Discrimination”) |
| Scopus | (hearing impairment*) OR “hearing loss” OR “hearing aid” OR “hearing aids” OR “hard of hearing” OR Presbycusis  AND  stigma* OR “social perception” OR “social perceptions” OR “Perceived Discrimination” |
| Embase Classic +Embase <1947 to 2024 April 28> | \| 1 \| exp hearing impairment/ \| 122741 \| \| --- \| --- \| --- \| \| 2 \| exp hearing aid/ \| 33041 \| \| 3 \| hearing impaired person/ \| 1558 \| \| 4 \| (hearing impairment* or "hearing loss" or "hearing aid" or "hearing aids" or "hard of hearing" or Presbycusis).mp. [mp=title, abstract, heading word, drug trade name, original title, device manufacturer, drug manufacturer, device trade name, keyword heading word, floating subheading word, candidate term word] \| 140919 \| \| 5 \| 1 or 2 or 3 or 4 \| 170289 \| \| 6 \| stigma/ or social stigma/ \| 26642 \| \| 7 \| (stigma* or "social perception" or "social perceptions" or "Perceived Discrimination").mp. [mp=title, abstract, heading word, drug trade name, original title, device manufacturer, drug manufacturer, device trade name, keyword heading word, floating subheading word, candidate term word] \| 70893 \| \| 8 \| 6 or 7 \| 70893 \| \| 9 \| 5 and 8 \| 363 \| |

# Supplemental Table 2. Characteristics of Studies on Stigma and Hearing Loss in Pediatric Settings (n=16)

| Author, Year | Journal | Study Design Instruments | Study Cohort(s) | Cohort Age(s) | Cohort Gender(s) | Measurement of Stigma | Stigma Related Findings | Quantitative Stigma Results | Themes |
| --- | --- | --- | --- | --- | --- | --- | --- | --- | --- |
| Hétu, 1996^1^ | Scandinavian Audiology  *Abstract Only* | Focus groups and interviews of children according to The DISABKIDS Questionnaires Handbook | *[1] Pediatric cohort with hearing loss*: (N=81)  *[2] Caregivers of children with hearing loss* (N=NR) | *Pediatric cohort with hearing loss*: Ages 8-18 years  *Caregiver cohort*: NR | *Pediatric cohort with hearing loss*: NR  *Caregiver cohort*: NR | Mention of perceived stigma in participant responses | 18.5% of responses were related to limitations in daily activities due to HAs and perceived stigma (e.g. exposure to others' curiosity) | NR | *Positive(s):*  Ease of use  *Negative(s):*  Visibility, exposure to classmates’ curiosity, bullying, lack of self-identification of disability, limitations in participation of daily activities |
| Blood 1997^2^ | Journal of Rehabilitation | Age matched peers without hearing loss and their HA preferences (n=100) regarding speech understanding, sound amplification, and cosmetics under hearing loss simulations | *[1] Age matched pediatric peers without hearing loss* (n=100) | *Age matched pediatric peers without hearing loss:* Ages 18-22 years | *Age matched pediatric peers without hearing loss:* 37M/63F | Subjects' responses before and after simulated conditions | After HA fitting, 25% of participants preferred not to wear BTE or ITE HAs. Subjects cited cosmetics and perceived stigma as primary reasons for not wearing HAs (69% mentioned visibility and size, 53% indicated perceived stigma). | NR | *Positive(s):* n/a  *Negative(s):*  Visibility, cosmetics |
| Jardine, 1999^3^ | The Journal of Laryngology & Otology | Questionnaire given to caregivers of children ages 4-11 years who were offered binaural HAs to manage otitis media with effusion | *[1] Caregivers of children with hearing loss* (n=39) | *Caregiver cohort*: NR | *Caregiver cohort*: NR | Thematic analysis of interview responses | 38 parents felt that the aids were easy to use. One parent did not use them for her child as she felt the stigma would be too great | NR | *Positive(s):*  Ease of use  *Negative(s):*  Visibility, exposure to classmates’ curiosity, bullying |
| Kent, 2003^4^ | Journal of Deaf Studies and Deaf Education | Survey of children with hearing loss (n=52) and their age matched peers without hearing loss (n=470) | *[1] Pediatric cohort with hearing loss*: (n=52)  *[2] Age matched pediatric peers without hearing loss* (n=470) | *Pediatric cohort with hearing loss*: Ages 11-15 years  *Age matched pediatric peers without hearing loss:* Ages 11-15 years | *Pediatric cohort with hearing loss*: 29M/23F  *Age matched pediatric peers without hearing loss:* 240M/230F | The Health Behavior in School-Aged Children (HBSC) questionnaire | Majority of HOH students (55.8%) did not self-identify as having a hearing disability. Willingness to self-identify was significantly associated with loneliness and bullying. Reluctance to self-identify may reflect the prevalence of negative stigma*.* | Mean scores of HBSC between HOH and hearing. There was a significant difference in loneliness scores for students who self-identified as HOH [4.13(1.60)] and students who did not [3.14(0.99)] | *Positive(s):*  positive feelings toward school/learning  *Negative(s):*  lack of self-identification of disability, bullying, loneliness |
| Ryan, 2006^5^ | Australian and New Zealand Journal of Audiology  *Abstract Only* | Age matched peers without hearing loss (n=NR) viewed pictures of children, with and without visible HAs, and rated their perceptions on an attitude scale | *[1] Age matched pediatric peers without hearing loss* (n=100) | *Age matched pediatric peers without hearing loss*: Ages 5 -12 years | *Age matched pediatric peers without hearing loss*: NR | Perceptions on an attitude scale. | Indigenous Australian children had more negative attitudes towards peers who wore HAs in comparison to peers who did not. Females tended to rate males more negatively than females on most questions. | NR | *Positive(s):* n/a  *Negative(s):*  Visibility, exposure to classmates’ curiosity, bullying |
| Kent, 2006^6^ | Journal of Deaf Studies and Deaf Education | Unstructured interviews conducted among adolescents with bilateral moderate to severe hearing loss (n=16) | *[1] Pediatric cohort with hearing loss*: (n=16) | *Pediatric cohort with hearing loss*: Ages 12–17 years | *Pediatric cohort with hearing loss*: 8M/8F | Thematic analysis of interview responses | Self-stigmatization or socially induced perceptions of “abnormality” are one response to the visibility of HAs. | NR | *Positive(s):*  self-empowerment  *Negative(s):*  Visibility, exposure to classmates’ curiosity, bullying |
| Strange, 2008^7^ | Australian and New Zealand Journal of Audiology  *Abstract Only* | Northern Territory Indigenous adolescents (n=NR) in boarding school shown  12 photos of male and female peers wearing a BTE HA, bone conduction HA, or no HA | *[1] Age matched pediatric peers without hearing loss* (n=NR) | *Age matched pediatric peers without hearing loss*: NR | *Age matched pediatric peers without hearing loss*: NR | Explored whether a 'HA Effect' (negative stigma associated with HAs) serves as the basis for lack of HA use | The more visible the HA, the more negatively the adolescents viewed the individual. A brief intervention explaining the benefits of HAs provided some desensitization to bias. | NR | *Positive(s):* n/a  *Negative(s)*:  Visibility, exposure to classmates’ curiosity, bullying |
| Meyer 2013^8^ | JAMA Otolaryngology Head Neck Surgery | Convenience sample of children with severe-to-profound SNHL surveyed with the Youth Quality of Life Instrument–Deaf and Hard of Hearing (YQoL-DHH) | *[1] Pediatric cohort with hearing loss*: (n=157) | *Pediatric cohort with hearing loss*: Ages 11-18 years | *Pediatric cohort with hearing loss*: 75M/82F | YQoL-DHH scores, which are composed of 3 domains: participation, self-acceptance, and stigma-related quality of life | When stratified by school setting, youths in mainstream schools with or without DHH programs using cochlear implants had higher participation, self-acceptance/advocacy, and stigma-related QoL scores compared with those using HAs. | Mean YQoL-DHH Scores, with stigma related QOL scores [mean (sd)] of 75.8 (16.5), 61.8 (22.8), and 72.4 (21.7) for no device, HA users, and CI users, respectively. | *Positive(s):*  self-empowerment  *Negative(s):*  Lack of environmental support |
| Tierney 2013^9^ | International Journal of Pediatric Otorhinolaryngology | Unstructured interviews among caregivers of children who had non-syndromic cleft palate about decision-making and satisfaction with ventilation tubes and/or HAs | *[1] Caregivers of children with hearing loss*  (n= 37) | *Caregiver cohort:* NR | *Caregiver cohort:* 4M/33F | Thematic analysis of interview responses | Parents expressed fears about the potential for social stigma of HAs. None reported significant teasing of children because they wore HAs. | NR | *Positive(s):*  ease of use  *Negative(s):*  Visibility, exposure to classmates’ curiosity, fear of rejection |
| Schick 2013^10^ | The Journal of Deaf Studies and Deaf Education | Standardized questionnaires including the YQoL-DHH conducted among youths with bilateral mild to profound hearing loss | *[1] Pediatric cohort with hearing loss*  (n=221) | *Pediatric cohort with hearing loss*: Ages 11–18 years | *Pediatric cohort with hearing loss*: 111M/110F | YQoL-DHH scores, which are composed of 3 domains: participation, self-acceptance, and stigma-related quality of life | In the Perceived Stigma domain, youths with normal hearing parents reported higher stigma in school for DHH placements than their peers with hard of hearing parents. | Mean (sd) perceived stigma scores among youths in schools for DHH with hearing parents [34.0 (18.7)] and with DHH parents [12.3 (13.5)] vs youths in schools w/o DHH support with hearing parents [24.9 (21.1)] and with DHH parents [35.5 (21.9)] | *Positive(s):* n/a  *Negative(s):*  exposure to classmates’ curiosity, bullying |
| Purcell 2016^11^ | International Journal of Pediatric Otorhinolaryngology | Youths with unilateral hearing loss (n=16) participated in hour-long interviews, parents (n=50) completed a 36-item survey | *[1] Pediatric cohort with hearing loss*: (N=16)  *[2] Caregivers of children with hearing loss* (N=50) | *[1] Pediatric cohort with hearing loss*: Ages 5–19 years  *[2] Caregivers of children with hearing loss:* NR | *[1] Pediatric cohort with hearing loss*: NR  *[2] Caregivers of children with hearing loss:* NR | Thematic analysis of interview responses | 12 of 16 (75%) stated that they had access to preferential seating in class but half reported difficulties with preferential seating, such as stigma and hesitancy to request a new seat. Among the 10 who had tried an FM system, 3 complained of stigma with using the device. 1 reported social stigma as the primary reason for cessation. Of the 4 who had never tried a device, 3 cited social stigma as the primary reason for not using one. | NR | *Positive(s)*:  positive feelings toward school/learning  *Negative(s)*:  visibility, exposure to classmates’ curiosity, fear of rejection |
| Chang 2017^13^ | Patient Education and Counseling | Semi-structured interviews among caregivers (n=33) of Deaf children ages 2-17 years coded using iterative and thematic coding | *[1] Caregivers of children with hearing loss*  (n= 33) | *Caregiver cohort:* NR | *Caregiver cohort:* 33F | Thematic coding of interview analyses | Some parents feel stigmatized and ostracized because they are a hearing parent who decided to get the cochlear implant(s) for their child(ren). | NR | *Positive(s):* n/a  *Negative(s):*  fear of rejection |
| Alvarenga, 2019^14^ | 26th Annual Conference of the International Society for Quality of Life Research  *Abstract Only* | Focus groups and interviews conducted among children and their caregivers in Brazil and Germany, based on European DISABKIDS and KIDSCREEN questionnaires | *[1] Pediatric cohort with hearing loss*: (n=81)  *[2] Caregivers of children with hearing loss:* (n= NR) | *[1] Pediatric cohort with hearing loss*: Ages 8-18 years  *[2] Caregiver cohort:* NR | *[1] Pediatric cohort with hearing loss*: NR  *[2] Caregiver cohort:* NR | Mention of perceived stigma in participant responses | Of the statements, 18.5% related to limitations in daily activities due to HAs e.g. during sports or in noisy environments, and perceived stigma e.g. exposure to others' curiosity | NR | *Positive(s):* n/a  *Negative(s):*  Visibility, exposure to classmates’ curiosity, limitations in participation of daily activities |
| Qian 2021^15^ | Laryngoscope | Adults and children without hearing loss surveyed on photos of a 10-year-old boy in six conditions with and without HAs and/or neutral or brightly colored glasses | *[1] Age matched pediatric peers without hearing loss* (n=202)  *[2] Unrelated (i.e. non-caregiver) adults without hearing loss* (n=210) | *[1] Age matched pediatric peers without hearing loss*: Age 10 years  *[2] Adult cohort:* Ages 18–65 years | *[1] Age matched pediatric peers without hearing loss*: 100M/100F    *[2] Adult cohort:* 105M/100F | Visual analogue scale (VAS) consisting of athleticism, confidence, friendliness, health, intelligence, leadership ability, popularity, and overall success in the future | HAs were associated with decreased athleticism, confidence, health, leadership, and popularity. When worn in combination with glasses, the beneficial effects of glasses were mitigated by brightly colored but not neutral HAs. | Mean differences in VAS scores reported | *Positive(s):* n/a  *Negative(s):*  Visibility, Exposure to classmates’ curiosity, bullying |
| Lin 2021^16^ | International Journal of Audiology | Semi-structured interviews with caregivers with children <3 years with mild, bilateral SNHL until saturation of themes was achieved | *[1] Caregivers of children with hearing loss:* (n= 12) | *[1] Caregiver cohort:* NR | *[1] Caregiver cohort:* 3M/9F | Perceived negatives of HA use including potential bullying/stigma. | Parental recorded statements of concern for their child(ren)’s mental/emotional safety in the face of HA stigma | NR | *Positive(s):*  self-empowerment  *Negative(s):*  Visibility, exposure to classmates’ curiosity, bullying |
| Jaradeh 2023^17^ | Otolaryngology – Head and Neck Surgery | Semi-structured interviews with immigrant and/or non-English speaking children with hearing loss and their caregivers | *[1] Pediatric cohort with hearing loss*: (n=532)  *[2] Caregivers of children with hearing loss:* (n= 12) | *[1] Pediatric cohort with hearing loss*: Average 11 years  *[2] Caregiver cohort*: NR | *[1] Pediatric cohort with hearing loss*: 270M/262  *[2] Caregiver cohort*: NR | Responses from semi-structured qualitative interviews | Foreign-born children with HL have significant delays in diagnosis and intervention compared to US-born children. For non-English-speaking parents, the diagnosis of HL presents challenges beyond that of the immigrant experience | NR | Positive(s): n/a  Negative(s): Visibility, fear of rejection, lack of environmental support |

Abbreviations: BTE, Behind-the-Ear; DHH, Deaf and hard of hearing; F, female; FM, frequency modulation; HA, hearing aid; HOH, hard of hearing; ITE, In-the-ear; M, male; NR, not reported; SD, standard deviation; SNHL, sensorineural hearing loss

#

#

# Supplemental Table 3. Characteristics of Studies on Stigma and Hearing Loss in Working Age Populations (n=19)

| Author, Year | Journal | Study Design | Cohort Age | Cohort Gender | Measurement of Stigma | Stigma Related Findings | Quantitative Stigma Results | Themes |
| --- | --- | --- | --- | --- | --- | --- | --- | --- |
| Hétu, 1990^18^ | British Journal of Audiology | [1] Group interviews with workers (n=NR) who participated in a pilot rehabilitation program and [2] Individual interviews (n=NR) conducted with hearing-impaired workers who have no prior contact with hearing specialists | NR | NR | Based on analysis of interview transcripts | Hearing impaired workers were strongly stigmatized by co-workers. The reluctance to acknowledge hearing difficulties was expressed through various forms of denial, minimization of the problem, uneasiness in talking about the problem | NR | *Positive(s)*: n/a  *Negative(s):* Visibility, fear of rejection, isolation from coworkers, peer perception of intelligence or adequacy |
| Brooks, 1994^19^ | British Journal of Audiology | Individuals (n=184) who had been fitted with BTE HAs and purchased ITE HAs were asked why they had purchased the ITE model | NR | 75M/86F | Questions targeting visibilities and other people's perception of HAs (i.e. senility, stupidity) | Reasons cited in favor of BTE over ITE: cosmetics acceptability, comfort, sound quality, and ease of use with glasses. | Ratings of ITE vs BTE reported, no other quantitative results | *Positive(s)*: n/a  *Negative(s):* ageism, peer perception of intelligence or adequacy |
| Hallberg, 1996^20^ | British Journal of Audiology | Unstructured interviews conducted among women (n=10) with noise-induced hearing loss | Ages 46-71 years | 10F | Stigma was one of the four categories grounded in the data from the interviews | Stigma was further categorized into: Negative attitudes, viewed as mentally retarded, prematurely aged, and as a deviating person. | NR | *Positive(s)*: n/a  *Negative(s):*  Ageism, peer perception of intelligence or adequacy |
| Garstecki, 2001^21^ | American Journal of Audiology | Three groups of women (total n=191) with self-reported normal hearing recruited by newspaper advertisements completed semantic differential statements related to hearing loss and HA use | Group 1: Ages 35-45 years (n=62);  Group 2: Ages 55-65 years (n=67);  Group 3: Ages75-85 years (n = 62) | 191F | Participants were asked to answer sentences ("If someone has a hearing loss, other people think of them as") with semantic differentials. (e.g., "Old vs. Young"). | Group differences for 6/7 semantic pairs: Dumb vs. Smart, Old vs. Young, Handicapped vs. Normal, and Unsuccessful vs. Successful, Cold vs. Warm, and Unfriendly vs. Friendly. In all cases, degree of negativity was inversely related to age. | Participants rated bipolar adjective scales on a scale from 1 to 7 (7 being positive) | *Positive(s)*: n/a  *Negative(s):*  Ageism, peer perception of intelligence or adequacy |
| Erler, 2002^22^ | American Journal of Audiology | Three groups of women (total n=191) with self-reported normal hearing recruited by newspaper advertisements completed semantic differential statements related to hearing loss and HA use | Group 1: Ages 35-45 years (n=62);  Group 2: Ages 55-65 years (n=67);  Group 3: Ages75-85 years (n = 62) | 191F | Participants were asked to answer sentences ("If someone has a hearing loss, other people think of them as") with semantic differentials. (e.g., "Old vs. Young"). | Negative perceptions associated with hearing loss and HA use are affected by age. Younger women perceive greater stigma than older women. Less stigma is associated with HA use than hearing loss, suggesting a positive effect of hearing loss management. | Participant answers scales on a scale from 1 to 7 (7 being positive) | *Positive(s)*: n/a  Negative(s):  Ageism, peer perception of intelligence or adequacy |
| Sankar, 2006^23^ | Genetics in Medicine | Qualitative interviews among individuals with hearing loss (n=23). | Ages 24-65 years | 6M/17F HOH | Interview responses | Subjects with hearing loss provided the most consistently positive comments about a genetic or hereditary basis to their condition. Comments about hearing loss from nongenetic causes were generally negative and focused on lack of acceptance. | NR | *Positive(s)*: n/a  *Negative(s):*  lack of family acceptance, limited intrafamilial communication |
| Kochkin, 2007^24^ | Hearing Journal | Two separate surveys: One mailed to 80,000 members of the National Family Opinion panel and the second sent to 3000 random adults with hearing loss who had not yet adopted HAs | Ages 56-62 years | NR | Dedicated follow-up survey asking about stigma and HAs | Non-adopter mean average ages are between 8 and 15 years lower than those of typical non-users. Of the non-adopters: Nearly half (48%) indicated that stigma contributed to their desire not to wear HAs out of concern for visibility and being perceived as old. | Tallies for each reason for HA non-use. No other quantitative results reported | *Positive(s)*: Improvement in hearing, positive impact on family and friends  *Negative(s):* Visibility, ageism, peer perception of intelligence or adequacy |
| Tye-Murray, 2009^25^ | Ear and Hearing | Participants with salaried, office-setting positions (n=48) participated in one of seven focus groups | Ages 29-79 years | 27M/21F | Analysis of interview transcripts | Unlike workers who have occupational hearing loss, this study population did not seem to experience an inordinate degree of stigmatization at the workplace, although most believe that hearing loss has negatively affected their performance. | NR | *Positive(s)*:  self-empowerment, locus of control  *Negative(s):* Visibility, fear of rejection, isolation from coworkers, peer perception of intelligence or adequacy |
| Southall, 2011^26^ | International Journal of Audiology | Semi-structured interviews conducted among 12 adults with adult-onset hearing loss and employed (n=12) designed to probe issues related to hearing loss disclosure | Ages 43-73 years | 6M/6F | Thematic analysis of interviews | Community affiliation was jeopardized because hearing loss was not considered ‘normal’. | NR | *Positive(s)*: Visibility of hearing aid took care of responsibility and burden of hearing loss disclosure to co-workers  *Negative(s):* Visibility, peer perception of intelligence or adequacy |
| Hindhede 2012^27^ | Health (London) | Face-to-face unstructured interviews with participants recently diagnosed with hearing loss (n=41) prior to and six weeks after HAs | Ages 23-70 years | 20M/21F | Thematic analysis of interview responses | Identification as a HA wearer does not relieve the individual from the stigma as the HA merely makes the disability visible. | NR | *Positive(s)*:  Quality of life, sense of normalcy  *Negative(s):* Visibility, peer perception of intelligence or adequacy, ageism |
| Preminger 2014^28^ | Ear and Hearing | Semi-structured interviews conducted among adults with hearing impairment (n=24) in four countries | Ages 26-96 years | 15M/19F | Thematic analysis of interview responses | Participants did not want to wear HAs because it would make them look older. Some younger participants wanted to differentiate themselves from older HA users. | NR | *Positive(s)*: Improvement in quality of life, reduced mental workload  *Negative(s):* Visibility, ageism, peer perception of intelligence or adequac |
| Skilton, 2016^12^ | International Health | Semi-structured interviews (n=21) conducted in Pokhara, Nepal during February-March 2014 | [1] Males ages 14–28 years  [2] Males ages 29–50 years  [3] Females ages 14-28 years  [4] Females ages 29-50 years | 11M/10F | Thematic analysis of semi-structured interviews | Stigma was both experienced and feared by participants throughout their lives, and as a result, they would not disclose their problem to friends, teachers and employers. Participants faced barriers in their education and work but did not receive the support they required because people were unaware of their condition. | NR | *Positive(s):* n/a  *Negative(s):* bullying, fear of rejection, lack of environmental support |
| Benito, 2016^29^ | Journal of Disability Policy Studies | Assess “hearing earnings gaps,” between individuals who are deaf or HOH and their normal hearing counterpart through Blinder–Oaxaca decompositions | Ages 25-40 years | 173,491M/ 135,605F | Income assessment | Roughly 40% of the overall hearing earnings gap is attributable to differences in educational attainment, potential experience, race/ethnicity, and marital status. The remaining 60% may reflect differences in communication skills and other unobservable characteristics (i.e. discrimination and stigma). | Yes, regarding income gaps but not for stigma | *Positive(s)*: n/a  *Negative(s):*  stigma may contribute to overall earnings gap experienced by adult HOH |
| Desjardins, 2017^30^ | American Journal of Audiology | Clinical trial of adults with mild to moderate bilateral SNHL who had never worn HAs were fitted with receiver-in-canal HAs for 6 weeks (n=24) with a control group (n=16) fitted without amplification | [1] Experimental group ages 50-74 years  [2] Control group ages 51–70 years | 15M/9F in experimental group | [1] HA Attitude Questionnaire (HARQ)  and  [2] International Outcome Inventory for HAs | Using HAs for 6 weeks significantly reduced participants' perceived stigma of HAs, personal distress and inadequacy due to hearing difficulties, and hearing handicap. | Statistically significant difference in mean HARQ score for HA stigma: 13 (2.7) before fitting, 10.5 (1.3) after 6 weeks, 10.4 (1.5) 2 weeks post-use. No significant difference in HARQ score for hearing loss stigma. | *Positive(s)*:  self-empowerment, improvement in quality of life  *Negative(s):* Visibility, ageism, peer perception of intelligence or adequacy |
| Lucas, 2018^31^ | International Journal of Audiology | Three group interviews conducted among adults (n=8) with moderately severe hearing loss using critical incident technique | Ages 37–71 years | 4M/4F | Inductive thematic analysis of interview responses | Participants felt that there was a “social stigma” about their hearing loss from others who lacked understanding and empathy. Participants also reported feelings of self-stigma (negative perception of oneself due to hearing loss) and low self-efficacy | NR | *Positive(s)*: n/a  *Negative(s):* Visibility, ageism, peer perception of intelligence or adequacy, low self-esteem |
| Michael, 2019^32^ | Rehabilitation Counseling Bulletin | 3 Questionnaires (YQoL-DHH, In Charge Financial Distress/Financial Well-Being scale, and a background questionnaire) conducted among CI users (n=30) and HA users (n=36). | Ages 21-74 years | 25M/41F | Youth Quality of Life–Deaf and Hard-of-Hearing (YQoL-DHH) adapted to an adult population | CI users reported higher levels of participation and lower levels of perceived stigma as compared with HA users | Perceived stigma, t(58) = −1.80, p < .05. | *Positive(s):* Improvement in quality of life, self-acceptance  *Negative(s):* Visibility, peer perception of intelligence or adequacy |
| Lash, 2020^33^ | Southern Communication Journal | Unstructured interviews conducted among participants with at least a moderate hearing loss  (n=30) by a HOH individual (primary researcher) | Ages 18-79 years | 18M/12F | Thematic analysis of how participants experienced or responded to stigma | Interviews identified five major stigmatizing experiences: feelings of sorry or pity, not feeling worth others’ time, being labeled as “not normal”, the perception that hearing loss limits capabilities and intelligence, and the idea that hearing loss is different from other types of disabilities. | NR | *Positive(s):* educational opportunity to educate others on hearing loss  *Negative(s):* Visibility, peer perception of intelligence or adequacy |
| Underdown, 2021^34^ | International Journal of Audiology | Semi-structured interviews with set open ended questions conducted among adutls with single sided deafness (n=8) | Ages 40-80 years | 2M/6F | Narrative and thematic analysis | No participant positively described the appearance of any of the interventions. For some, HA appearances were a factor in rejecting them. | NR | *Positive(s)*: Improved hearing, improved quality of life  *Negative(s):* Visibility, ageism, peer perception of intelligence or adequacy |
| Archana, 2016^35^ | Indian Journal of Otology | Cross-sectional survey of adults with bilateral moderate hearing loss or prior monaural or binaural HA fitting  (n=100) | Ages 41–65 years | 52M/48F | Participants answered “yes” (2), “no” (0), or “sometimes” (1) when asked if appearance and psychosocial reasons contributed to HA nonuse | Most female respondents reported appearance factor as the most common cause for rejection of the HA (not statistically significant) | Mean(sd) scores 2.00 (1.12) and 1.88 (1.15) among females and males, respectively | *Positive(s):* n/a  *Negative(s):*  fear of rejection |

Abbreviations: CI, Cochlear implant; F, female; FM, frequency modulation; HA, hearing aid; HOH, hard of hearing; ITE, In-the-ear; M, male; NR, not reported; SD, standard deviation; SNHL, sensorineural hearing loss

# Supplemental Table 4. Characteristics of Studies on Stigma and Hearing Loss in Older Adults (n=14)

| Author, Year | Journal | Study Design | Cohort Age | Cohort Gender | Measurement of Stigma | Stigma Related Findings | Quantitative Stigma Results | Themes |
| --- | --- | --- | --- | --- | --- | --- | --- | --- |
| VandenBrink, 1996^36^ | British Journal of Audiology | Survey data among hearing impaired (n=114) using a revised Health Belief Model | Ages ≥ 57 years | 50M/64F | Survey included beliefs about the severity of the hearing problems, the benefits of a HA, barriers to HA use, and the opinions of significant others | Non-help seekers perceived impairment as relatively inconsequential and demonstrated a passive acceptance of hearing problems. Those who did not try an aid after consulting their doctor reported the most stigma-related barriers to HA use and felt their significant others agreed with them. Current users had the most favorable attitude toward HAs. | NR | *Positive(s)*: self-empowerment, improved hearing, improved quality of life  *Negative(s)*: ageism, visibility, fear of rejection, acceptance of disability |
| Brooks, 1998^37^ | British Journal of Audiology | First time HA candidates (n = 135) were administered the Hearing Attitudes in Rehabilitation Questionnaire (HARQ) before and 3-9 months after HA fitting | Mean (SD) 74 (10) years | NR | Stigma is a subscale of the HARQ | An attitude that wearing a HA was stigmatizing was significantly associated with reporting that HAs were difficult to handle in logistic regression | OR of reporting HA difficulty 3.48, p=0.03  with reported aid stigma | *Positive(s)*: n/a  *Negative(s)*: visibility, fear of rejection |
| Garstecki, 1998^38^ | Journal of Speech, Language, and Hearing Research | Questionnaire administered to adults >65 years with adult-onset hearing loss subsequently assessed and recommended for HAs (n=131) | Female adherents (n=35, ages 67-84 years)  Female non-adherents (n=33, ages 65-90 years)  Male adherents (n=25, ages 66-90 years)  Male non-adherents (n=38, ages 65-85 years) | 68F/63M | HA questionnaire covering four domains (HA comfort/benefit, public reaction, costs, and support from friends and family) | Although public reaction, or stigma, was rated as the least important factor related to HA use, it was of greater concern to non-adherents, particularly men. | NR | *Positive(s)*: n/a  *Negative(s)*: visibility, self-perception of weakness |
| Doggett, 1998 ^39^ | Journal of the American Academy of Audiology | Women (n=20) rated an aided and unaided peer after reading to them. Raters were separated into two groups: informed judges were told that the aided  peers were wearing HAs, whereas the  uninformed judges were not | Ages 60-91 years | 20F | Perceived age and personality attributes on a Likert scale | Judges perceived their aided peers significantly more negatively than their unaided peers on  confidence, intelligence, and friendliness. | Differences in perceived confidence (N=9, R= 8.0, p<.05), intelligence (N = 6, R = 0, p<.05), and friendliness (N = 6, R = 0, p<.05) | *Positive(s)*: n/a  *Negative(s)*:  fear of rejection, peer perception of intelligence or adequacy |
| Meister, 2008^40^ | International Journal of Audiology | Questionnaire study assessing pre-fitting expectations and willingness to use HAs among hearing aid candidates (n=100) | Ages 32-92 years (median 70 years) | 52M/48F | Agreement [1 (very high) – 5 (very low)] to the following statement: “HAs are not negatively perceived by others (e.g. by giving the impression of being old or inadequate)” | Subjects who admit stigmatization tend to avoid HA provision. However, it was interesting that expectation towards stigmatization was less powerful with respect to the model than the expectation that HAs have a positive effect on quality of life | Mean (SD) 2.9 (1.1) | *Positive(s)*: improvement in quality of life  *Negative(s)*: ageism, peer perception of intelligence or adequacy |
| Southall, 2010^41^ | International Journal of Audiology | Audio-recorded Semi-structured interviews of adults with hearing loss and were members of peer-support groups (N=10) | Mean 65 years | 3M/7F | Thematic analyses of interview responses | Lasting decisions about hearing loss management were made following ‘critical junctures’ (i.e. negative psychosocial stresses far outweighed positive feelings, or when the positive outlook far outweighed the negative stress) | NR | *Positive(s)*:  self-empowerment, community  *Negative(s)*: visibility, peer perception of intelligence or adequacy, fear of rejection |
| Wallhagen, 2010^42^ | Gerontologist | Semi-structured interviews conducted over 1 year with dyads where one partner had hearing loss (n=91 dyads) | Ages >60 years | 52M/39F among hearing impaired participants | Analysis of interview responses with grounded theory and constant comparative methodology | Stigma was related to 3 interrelated experiences, alterations in self-perception, ageism, and vanity and was influenced by dyadic relationships and external societal forces, such as health and hearing professionals and media. | NR | *Positive(s)*: improvement in quality of life  *Negative(s)*: ageism, visibility, cosmesis, self-perception of weakness |
| Wanstrom, 2014^43^ | Hearing, Balance and Communication | Semi-structured interviews among participants in Sweden with acquired sensorineural hearing impairment (n=18) | Ages 50-70 years | 11M/7F | Thematic analysis of interview responses | Participants described the process of acceptance as involving their social network of family, friends and colleagues. They noted a trade-off between the consequences of untreated hearing impairment and the stigma of hearing impairment. | NR | *Positive(s)*:  ease of access, self-empowerment  *Negative(s):*  Visibility, peer perception of intelligence or inadequacy |
| Rolfe, 2016^44^ | International Journal of Audiology | Semi-structured interviews with thematic analysis (n=22) | Ages 66-88 years | 9M/13F | Thematic analysis of interviews | The visibility of HAs delayed participants getting help, and many expressed a strong preference for hidden HAs. Ironically, HA marketing aimed at increasing uptake by emphasizing that HAs can be hidden was viewed by some as perpetuating stigma, and some felt that enhancing HA visibility could destigmatize HAs and HL. | NR | *Positive(s)*: n/a  *Negative(s)*:  lack of self-identification of disability, ageism, peer perception of intelligence or inadequacy, visibility, cosmesis |
| Pronk, 2017^45^ | Trends in Hearing | Telephone questionnaire administered at baseline and 4 months later to determine if participants entered a HA Evaluation Period (HAEP) | Ages ≥55 years | 70M/59F | Stigma was measured using the six-item Stigma subscale. Scores can range from 6 to 30 (higher scores indicate greater stigma). | HA stigma and hearing loss severity were modified by gender such that they were only predictive of entering a HAEP for women and not for men. | Multivariate OR of stigma with entering a HA evaluation period: OR 1.04 (1.00–1.14) | *Positive(s)*: community  *Negative(s)*:  lack of self-identification of disability |
| David, 2018^46^ | American Journal of Audiology | Semi structured interviews were conducted among older persons with age-related hearing loss (n=11) | Ages ≥65 years | 4M/7F | Thematic analysis of interviews | 3 core dimensions of stigma identified: [1] cognitive attributions (being old, stupid, crippled); [2] emotional reactions (shame, pity, and feeling ridiculed); and [3] behavioral (concealment, distancing, HA adoption) | NR | *Positive(s)*: n/a  *Negative(s)*:  self-perception of weakness, fear of rejection, peer perception of intelligence or inadequacy |
| McKee, 2019^47^ | Gerontologist | Semi structured interviews (n=21) with community participants | Ages >55 years | NR | Thematic analysis of interviews | “No, it’s embarrassing. It’s like, I’m getting old. (Participant 19) “. I just turned 60. I’m not mentally ready for one, but I know I have a problem with hearing. (Participant 5) | NR | *Positive(s)*: n/a  *Negative(s)*: ageism, fear of rejection |
| McMahon, 2021^48^ | Public Health Research & Practice | Semi-structured focus groups conducted among participants from US, Australia, Canada, and UK with hearing status ranging from normal hearing to hearing problems with and without HA use (n=47) | Ages >60 years | 25M/22F | Thematic analysis of interviews | There were three  inter-related sources of stigma: having hearing loss, wearing HAs, and aging. Perceived barriers, facilitators and preferences were largely consistent across countries, with stigma and trust in hearing healthcare being the barriers most often discussed. | NR | *Positive(s)*: n/a  *Negative(s)*: ageism, self-perception of weakness, lack of environmental support |
| Pornprasit 2023^49^ | Frontiers in Digital Health | Focus group-based, purely qualitative design | Ages 60-88 years | 4M/22F | Thematic analysis of focus groups, phenomenological approach | Pivotal role of familial and social networks in mitigating barriers associated with hearing difficulties among older adults in Thailand | NR | *Positive(s)*: n/a  *Negative(s)*: ageism, self-perception of weakness, lack of self-identification of disability |

Abbreviations: BTE, Behind-the-Ear; F, female; FM, frequency modulation; HA, hearing aid; HAEP, hearing aid evaluation period; HOH, hard of hearing; ITE, In-the-ear; M, male; NR, not reported; OR, odds ratio; SD, standard deviation; SNHL, sensorineural hearing loss;

#

**Supplemental Table 5. Breakdown of International Studies**

| **Pediatric (n=9)** | **Working Age (n= 10)** | **Older Adults (n=10)** |
| --- | --- | --- |
| Hetu 1996 (Scandinavia) | Hetu 1990 (United Kingdom) | VandenBrink 1996 (United Kingdom) |
| Jardine 1999 (United Kingdom) | Brooks 1994 (United Kingdom) | Brooks 1998 (United Kingdom) |
| Kent 2003 (New Zealand) | Hallberg 1996 (United Kingdom) | Meister 2008 (Germany) |
| Ryan 2006 (Australia) | Southall 2011 (Canada) | Southall 2010 (Canada) |
| Strange 2008 (New Zealand) | Hindhede 2012 (Denmark) | Wänström 2014 (Sweden) |
| Tierney 2013 (United Kingdom) | Preminger 2014 (Australia, Denmark, United Kingdom, and US | Rolfe 2016 (United Kingdom) |
| Skilton 2016 (Nepal) | Lucas 2018 (United Kingdom) | Pronk 2017 (Netherlands) |
| Alvarenga 2019 (Brazil and Germany) | Michael (Israel) | Zoizner 2018 (Israel) |
| Lin 2021 (Australia) | Underdown 2021 (United Kingdom) | McMahon 2021(United Kingdom, Australia, Canada, US) |
| *Jaradeh 2023* (US, among non-english speaking immigrants)* | Archana 2016 (India) | Pornprasit 2024 (Thailand) |

# References

1. Hétu R. The Stigma Attached to Hearing Impairment. *Scand Audiol Suppl*. 1996;43:12-24. https://pubmed.ncbi.nlm.nih.gov/8738641/. Accessed May 15, 2022.

2. Blood IM. The hearing aid effect: Challenges for counseling. *J Rehabil*. 1997;63(4):59. https://www.proquest.com/docview/236267874. Accessed August 27, 2022.

3. Jardine AH, Griffiths M V., Midgley E. The acceptance of hearing aids for children with otitis media with effusion. *J Laryngol Otol*. 1999;113(4):314-317. doi:10.1017/S0022215100143865

4. Kent BA. Identity Issues for Hard-of-Hearing Adolescents Aged 11, 13, and 15 in Mainstream Setting. *J Deaf Stud Deaf Educ*. 2003;8(3):315-324. doi:10.1093/DEAFED/ENG017

5. Ryan BJ, Johnson A, Strange A, Yonovitz A. The “hearing aid effect” in northern territory indigenous Australian children as perceived by their peers. *Aust New Zeal J Audiol*. 2006;28(2):55-74. doi:10.1375/AUDI.28.2.55

6. Kent B, Smith S. They Only See It When the Sun Shines in My Ears: Exploring Perceptions of Adolescent Hearing Aid Users. *J Deaf Stud Deaf Educ*. 2006;11(4):461-476. doi:10.1093/DEAFED/ENJ044

7. Strange A, Johnson; A, Ryan B-J, Yonovitz A. The Stigma of Wearing Hearing Aids in an Adolescent Aboriginal Population. *Aust New Zeal J Audiol*. 2008;30(1):19-37. https://search.informit.org/doi/10.3316/INFORMIT.232570468267883. Accessed August 27, 2022.

8. Meyer A, Sie K, Skalicky A, et al. Quality of life in youth with severe to profound sensorineural hearing loss. *JAMA Otolaryngol Head Neck Surg*. 2013;139(3):294-300. doi:10.1001/JAMAOTO.2013.35

9. Tierney S, O’Brien K, Harman NL, Madden C, Sharma RK, Callery P. Risks and benefits of ventilation tubes and hearing aids from the perspective of parents of children with cleft palate. *Int J Pediatr Otorhinolaryngol*. 2013;77(10):1742-1748. doi:10.1016/J.IJPORL.2013.08.006

10. Schick B, Skalicky A, Edwards T, Kushalnagar P, Topolski T, Patrick D. School Placement and Perceived Quality of Life in Youth Who Are Deaf or Hard of Hearing. *J Deaf Stud Deaf Educ*. 2013;18(1):47-61. doi:10.1093/DEAFED/ENS039

11. Purcell PL, Jones-Goodrich R, Wisneski M, Edwards TC, Sie KCY. Hearing devices for children with unilateral hearing loss: Patient- and parent-reported perspectives. *Int J Pediatr Otorhinolaryngol*. 2016;90:43-48. doi:10.1016/J.IJPORL.2016.08.029

12. Skilton MK, Poole N, Metcalfe CW, Martin TPC, Smith MCF. The impact of ear disease and hearing impairment on the lives of Nepali patients in Pokhara: a qualitative study. *Int Health*. 2016;8(2):101-107. doi:10.1093/INTHEALTH/IHV052

13. Chang PF. Breaking the sound barrier: exploring parents’ decision-making process of cochlear implants for their children. *Patient Educ Couns*. 2017;100(8):1544-1551. doi:10.1016/J.PEC.2017.03.005

14. Alvarenga Reis R, Bullinger M, Borozan O, Brutt A. (1090) Identification of attributes of quality of life among children and adolescents with hearing loss. *26th Annu Conf Int Soc Qual Life Res*. 2019;28(Suppl 1):S1–S190. https://www.proquest.com/docview/2284300487?accountid=41157&parentSessionId=N1KftBS5kuFCktV9a8yVRJBEtdZ%2FymzCAipHDjjO7QY%3D&pq-origsite=primo. Accessed August 27, 2022.

15. Qian ZJ, Nuyen BA, Kandathil CK, et al. Social Perceptions of Pediatric Hearing Aids. *Laryngoscope*. 2021;131(7):E2387-E2392. doi:10.1002/LARY.29369

16. Lin JJ, Gillam L, Smith L, et al. Mild matters: parental insights into the conundrums of managing mild congenital hearing loss. *Int J Audiol*. 2021. doi:10.1080/14992027.2021.1954248

17. Jaradeh K, Liao EN, Chehab LZ, et al. Understanding Barriers to Timely Diagnosis and Intervention Among Immigrant Children With Hearing Loss. *Otolaryngol Head Neck Surg. 2023*;169(3):710-718. doi: 10.1002/ohn.322

18. Hétu R, Riverin L, Getty L, Lalande NM, St-Cyr C. The reluctance to acknowledge hearing difficulties among hearing-impaired workers. *Br J Audiol*. 1989;24(4):265-276. doi:10.3109/03005369009076565

19. Brooks DN. Some factors influencing choice of type of hearing aid in the UK: Behind-the-ear or in-the-ear. *Br J Audiol*. 2009;28(2):91-98. doi:10.3109/03005369409077919

20. Hallberg LRM, Jansson G. Women with noise-induced hearing loss: An invisible group? *Br J Audiol*. 2009;30(5):340-345. doi:10.3109/03005369609076782

21. Garstecki DC, Erler SF. Personal and social conditions potentially influencing women’s hearing loss management. *Am J Audiol*. 2001;10(2):78-90. doi:10.1044/1059-0889(2001/007)

22. Erler SF, Garstecki DC. Hearing loss- and hearing aid-related stigma: perceptions of women with age-normal hearing. *Am J Audiol*. 2002;11(2):83-91. doi:10.1044/1059-0889(2002/020)

23. Sankar P, Cho MK, Wolpe PR, Schairer C. What is in a cause? Exploring the relationship between genetic cause and felt stigma. *Genet Med*. 2006;8(1):33. doi:10.1097/01.GIM.0000195894.67756.8B

24. Kochkin S. MarkeTrak VII: Obstacles to adult non-user adoption of hearing aids. *Hear J*. 2007;60(4):24-51. doi:10.1097/01.HJ.0000285745.08599.7F

25. Tye-Murray N, Spry JL, Mauzé E. Professionals with hearing loss: Maintaining that competitive edge. *Ear Hear*. 2009;30(4):475-484. doi:10.1097/AUD.0B013E3181A61F16

26. Southall K, Jennings MB, Gagné JP. Factors that influence disclosure of hearing loss in the workplace. *Int J Audiol*. 2011;50(10):699-707. doi:10.3109/14992027.2011.588963

27. Hindhede AL. Negotiating hearing disability and hearing disabled identities. *Health (Irvine Calif)*. 2012;16(2):169-185. doi:10.1177/1363459311403946

28. Preminger JE, Laplante-Lévesque A. Perceptions of age and brain in relation to hearing help-seeking and rehabilitation. *Ear Hear*. 2014;35(1):19-29. doi:10.1097/AUD.0B013E31829C065C

29. Benito SG, Glassman TS, Hiedemann BG. Disability and Labor Market Earnings: Hearing Earnings Gaps in the United States. *J Disabil Policy Stud*. 2016;27(3):178-188. doi:10.1177/1044207316658752

30. Desjardins JL, Doherty KA. Changes in Psychosocial Measures After a 6-Week Field Trial. *Am J Audiol*. 2017;26(2):119. doi:10.1044/2017_AJA-16-0066

31. Lucas L, Katiri R, Kitterick PT. The psychological and social consequences of single-sided deafness in adulthood. *Int J Audiol*. 2018;57(1):21-30. doi:10.1080/14992027.2017.1398420

32. Michael R, Attias J, Raveh E. Perceived Quality of Life Among Adults With Hearing Loss: Relationships With Amplification Device and Financial Well-Being: *Rehabil Couns Bull*. 2017;62(4):234-242. doi:10.1177/0034355217738717

33. Lash BN, Helme DW. Managing Hearing Loss Stigma: Experiences of and Responses to Stigmatizing Attitudes & Behaviors. *South Commun J*. 2020;85(5):302-315. doi:10.1080/1041794X.2020.1820562

34. Underdown T, Pryce H. How do patients decide on interventions for single sided deafness? A qualitative investigation of patient views. *Int J Audiol*. 2021;61(7):551-560. doi:10.1080/14992027.2021.1951853

35. Archana G, Krishna Y, Shiny R. Reasons for nonacceptance of hearing aid in older adults. *Indian J Otol*. 2016;22(1):19. doi:10.4103/0971-7749.176513

36. Van den Brink RHS, Wit HP, Kempen GIJM, Van Heuvelen MJG. Attitude and help-seeking for hearing impairment. *Br J Audiol*. 1996;30(5):313-324. doi:10.3109/03005369609076779

37. Brooks DN, Hallam RS. Attitudes to hearing difficulty and hearing aids and the outcome of audiological rehabilitation. *Br J Audiol*. 1998;32(4):217-226. doi:10.3109/03005364000000069

38. Garstecki DC, Erler SF. Hearing loss, control, and demographic factors influencing hearing aid use among older adults. *J Speech Lang Hear Res*. 1998;41(3):527-537. doi:10.1044/JSLHR.4103.527

39. Doggett S, Stein R. Hearing Aid Effect in Older Females. *J Am Acad Audiol*. 1998;9(5):361-366. https://pubmed.ncbi.nlm.nih.gov/9806409/. Accessed August 27, 2022.

40. Meister H, Walger M, Brehmer D, Von Wedel UC, Von Wedel H. The relationship between pre-fitting expectations and willingness to use hearing aids. *Int J Audiol*. 2008;47(4):153-159. doi:10.1080/14992020701843111

41. Southall K, Gagné JP, Jennings MB. Stigma: a negative and a positive influence on help-seeking for adults with acquired hearing loss. *Int J Audiol*. 2010;49(11):804-814. doi:10.3109/14992027.2010.498447

42. Wallhagen MI. The stigma of hearing loss. *Gerontologist*. 2010;50(1):66-75. doi:10.1093/GERONT/GNP107

43. Wänström G, Öberg M, Rydberg E, Lunner T, Laplante-Lévesque A, Andersson G. The psychological process from avoidance to acceptance in adults with acquired hearing impairment. *Hear Balanc Commun*. 2014;12(1):27-35. doi:10.3109/21695717.2013.875243

44. Rolfe C, Gardner B. Experiences of hearing loss and views towards interventions to promote uptake of rehabilitation support among UK adults. *Int J Audiol*. 2016;55(11):666. doi:10.1080/14992027.2016.1200146

45. Pronk M, Deeg DJH, Versfeld NJ, Heymans MW, Naylor G, Kramer SE. Predictors of Entering a Hearing Aid Evaluation Period: A Prospective Study in Older Hearing-Help Seekers. *Trends Hear*. 2017;21. doi:10.1177/2331216517744915

46. David D, Zoizner G, Werner P. Self-Stigma and Age-Related Hearing Loss: A Qualitative Study of Stigma Formation and Dimensions. *Am J Audiol*. 2018;27(1):126-136. doi:10.1044/2017_AJA-17-0050

47. Mckee MM, Choi HJ, Wilson S, et al. Determinants of Hearing Aid Use Among Older Americans With Hearing Loss. *Gerontologist*. 2019;59(6):1171-1181. doi:10.1093/GERONT/GNY051

48. McMahon CM, Mosley CL, Pichora-Fuller MK, et al. Older adults’ perceptions of current and future hearing healthcare services in Australia, England, US and Canada. *Public Heal Res Pract*. 2021;31(5). doi:10.17061/PHRP3152128

49. Pornprasit P, Utoomprurkporn N, Areekit P, et al. Attitudes toward hearing difficulties, health-seeking behaviour, and hearing aid use among older adults in Thailand. *Front Digit Health.* 2024;10:5:1075502. doi: 10.3389/fdgth.2023.1075502
